# Dietary Patterns of Docosahexaenoic Acid Intake and Supplementation from Pregnancy Through Childhood with a Focus on Low- and Middle-Income Countries: A Narrative Review of Implications for Child Health

**DOI:** 10.3390/nu17243931

**Published:** 2025-12-16

**Authors:** Brenda Valle-Valdez, Xochitl Terrazas-Lopez, Alejandra Gonzalez-Rocha, Humberto Astiazaran-Garcia, Brianda Armenta-Guirado

**Affiliations:** 1 Department of Health Sciences, University of Sonora, Sonora 85010, Mexico; brenda.valle@unison.mx (B.V.-V.); a218218415@unison.mx (X.T.-L.); brianda.armenta@unison.mx (B.A.-G.); 2 Social Interventions Research and Evaluation Network, University of California, San Francisco, CA 94143, USA; alejandra.gonzalezrocha@ucsf.edu; 3 Department of Chemical and Biological Sciences, University of Sonora, Hermosillo 83000, Mexico; humberto.astiazaran@unison.mx; 4 Research Center for Food and Development (CIAD) A.C., Hermosillo 83304, Mexico

**Keywords:** docosahexaenoic acid (DHA), dietary intake, supplementation, health, childhood, low- and middle-income countries

## Abstract

Docosahexaenoic acid (DHA) is a long-chain omega-3 fatty acid essential for neurodevelopment, immune regulation, and key physiological functions during early life. In low- and middle-income countries (LMICs), limited access to DHA-rich foods contributes to disparities in intake and health outcomes. This narrative review describes the current evidence on dietary patterns of DHA intake and supplementation from pregnancy through childhood in LMICs and highlights the implications of these patterns for child health. The review is based on a systematic search conducted in PubMed using Medical Subject Heading (MeSH) terms related to DHA, dietary patterns, health outcomes, and LMICs. Studies published between 2014 and 2025 were screened using Covidence software. Eligible studies included observational, interventional, and review designs that reported DHA through dietary intake, supplementation, or measurement in biological samples during pregnancy, lactation, infancy, or childhood. Data extraction followed the PICOS (Population, Intervention, Comparison, Outcome, Study Design) framework. A total of 76 studies were included. Across LMICs, DHA intake was consistently insufficient among pregnant and lactating women, infants, and children. Reported dietary sources were generally low in DHA content. Intake or supplementation was associated with neurodevelopment, immune response, pregnancy outcomes, and cardiometabolic health, although findings were sometimes mixed or modified by gene–environment interactions. Results varied by study design, contextual factors, income level, and geographic access. Large gaps remain in nationally representative intake data. Despite its physiological relevance, DHA intake remains inadequate in LMICs during early life. This review underscores the importance of improving DHA intake in vulnerable populations and identifies evidence gaps to guide future research and inform context-specific nutrition strategies.

## 1. Introduction

Docosahexaenoic acid (DHA, 22:6*n*-3) is an indispensable type of long-chain polyunsaturated fatty acid (LCPUFA) with important structural and biochemical contributions across multiple life cycle stages. Its function is particularly pertinent in childhood, as it is a core component of neuronal membranes and modulates synaptic plasticity, neurotransmission, and gene expression. Sustained and intensive accumulation of DHA occurs during the third trimester of pregnancy and the first 2 years of life, periods when an exogenous supply is important, given limited endogenous synthesis capacity [1,2,3,4]. As mammals cannot synthesize DHA de novo, it must be obtained either directly from fish- and animal-based foods or indirectly through the inefficient conversion of alpha-linolenic acid (ALA). Plant oils and plant-based foods do not contain preformed DHA, making fish, seafood, and eggs the main dietary contributors [5]. Higher maternal DHA availability during the fetal and early childhood period has been associated with more positive neurodevelopmental outcomes, including Intelligence Quotient (IQ), language, visual–motor coordination, and attention [6,7]. Additionally, DHA may have key immunomodulatory functions, facilitating the resolution of inflammatory outcomes via specialized mediators, including resolvins, protectins, and maresins, and decreasing the expression of proinflammatory cytokines, including IL-6 and Tumor necrosis factor (TNF)-α, that are especially relevant over the course of immunological maturation during the childhood years [6,8].

While DHA has a well-established physiological role in many health outcomes throughout life, no Dietary Reference Intake has been specifically established for DHA. The Institute of Medicine (IOM) has set an Adequate Intake for ALA, a precursor of DHA, of 1.6 g/day and 1.1 g/day for men and women, respectively, suggesting that up to 10% of this intake should be composed of eicosapentaenoic acid (EPA) and DHA [9]. However, the endogenous conversion of ALA to DHA is highly inefficient, with most studies reporting rates lower than 1%, with a range between 0 and 9.2%, depending on methodological approaches. Although isotope dilution studies likely underestimate actual DHA synthesis, and other methods suggest that conversion may occur at rates higher than currently assumed, this process remains insufficient to meet physiological requirements, highlighting the necessity of dietary DHA sources [10,11]. The National Institutes of Health recommends 100 mg of DHA per day for newborns aged 0–12 months, emphasizing the essential function of DHA in early growth and development [11]. Similarly, the World Health Organization (WHO) and the European Food Safety Authority advise adults to consume a minimum of 250 mg of DHA + EPA per day, with pregnant and lactating women being advised to consume at least 200 mg of DHA per day to support optimal maternal and infant health [9,12]. Despite these recommendations, the average intake of DHA + EPA in high-income countries (HICs) such as the United States is approximately 100 mg daily [9], which is below international standards and underlines the need for life-stage-specific DHA intake recommendations tailored to the requirements of different population groups.

In addition to the lack of evidence to establish DHA requirements at different life stages, significant disparities in DHA access and consumption have been documented according to national income levels, with dietary DHA intake in Low- and Middle-Income Countries (LMICs) compared to HICs being low, possibly reflecting the limited availability of marine food sources, as well as a lack of geographic access, purchasing power, and public policies [13,14]. These dietary inequalities are particularly pronounced at life-cycle stages where there is an elevated physiological requirement for DHA (e.g., pregnancy, lactation, and infancy). Moreover, a recent concern has been reported regarding the impact of climate change on the availability of DHA in aquatic systems. The production of *n*-3 LCPUFA at the lower levels of the marine food chain could be affected by changes in water temperature or phytoplankton composition. Under a climate scenario that was recently modeled (2010–2100 y), global DHA availability is predicted to decline, with decreasing rates observed across regions in the coming decades [15], which could indicate a reduction in nutritional sufficiency in populations that use aquatic dietary sources as their main source of food.

Although multiple narrative reviews, systematic reviews, and meta-analyses have examined DHA intake and health outcomes [6,16,17,18,19,20,21,22], to our knowledge, no prior narrative review has focused on both dietary DHA intake and supplementation from pregnancy through childhood within the context of LMICs. This perspective is essential to identify knowledge gaps and structural inequities in DHA consumption across the life course, particularly among vulnerable populations, contribute to the recognition of DHA intake deficiency as a relevant public health problem, and highlight the need to generate evidence to support population-specific recommendations. In this context, the present narrative review aims to synthesize current evidence on dietary DHA intake and supplementation from pregnancy through childhood in LMICs, summarize how these patterns compare with those reported in HICs, and outline the implications of these disparities for maternal and child health, based on studies published between 2014 and 2025.

## 2. Materials and Methods

This review was conducted as a narrative review. We performed a structured search in PubMed for the years 2014–2025 using Medical Subject Headings (MeSH) terms related to “docosahexaenoic acid (DHA)”, “health”, and “dietary patterns”. To specifically retrieve studies from LMICs, we also used the MeSH term “Developing Countries”. Although the main focus was LMICs, studies from HICs were considered when they provided relevant contrasts or contextual information. Reference lists of eligible articles and reviews were also screened to identify additional studies. Covidence software [23] was used to remove duplicates, screen titles and abstracts, and facilitate article selection and data extraction. Two authors (BA-G and BV-V) independently screened titles and abstracts; in cases of discordance, a third author (AG-R) resolved the decision. Full-text articles were reviewed and selected by consensus among three authors (BA-G, BV-V, and XT-L). Eligible studies reported DHA through dietary intake, supplementation, or measurement in biological samples (e.g., red blood cells, plasma, cord blood, colostrum) during pregnancy, lactation, infancy, or childhood, defined according to WHO criteria [24]. To ensure transparency in the organizational process, Supplementary Table S1 was developed as a classification framework to group DHA-related outcomes identified across studies and to document this methodological step. Countries were classified by income level according to World Bank 2023 criteria, detailed in Supplementary Table S2.

## 3. Global DHA Intake

### 3.1. Comparison by Country Income Level

A total of 2293 studies were identified in the PubMed database, and 53 additional references were obtained by citation searching. After removing 84 duplicates, 2262 records were screened, of which 112 were screened for eligibility. Of these, 36 did not meet the inclusion criteria, primarily due to inappropriate route of administration (*n* = 5) or the wrong time (*n* = 6). Consequently, 76 studies were included in this narrative review (Supplemental Figure S1) as follow: 24 cross-sectional [25,26,27,28,29,30,31,32,33,34,35,36,37,38,39,40,41,42,43,44,45,46,47,48], 16 clinical trials [49,50,51,52,53,54,55,56,57,58,59,60,61,62,63,64], 14 reviews [1,3,4,6,8,13,20,22,65,66,67,68,69,70], 12 cohorts [2,71,72,73,74,75,76,77,78,79,80,81], 6 systematic review [16,18,82,83,84,85], 3 ecological [86,87,88], and 1 meta-analysis [17].

Estimating DHA intake globally remains a challenge due to the lack of consistent and systematic information with national representation from all countries around the world. In this regard, we identified only one study that provides a worldwide estimation of DHA intake [86]; however, within the scope of this review, we did not find any studies specifically reporting on DHA dietary intake or consumption in children at the global level.

Global estimates of DHA intake remain limited, with the ecological study by Forsyth et al. [86] providing the most comprehensive analysis to date. Using the Food and Agriculture Organization of the United Nations (FAO) Food Balance Sheets (FBS), median per capita intake across 172 countries showed marked disparities by income group: 192 mg/day (range: 67–706 mg) in high-income countries (*n* = 42), 122 mg/day (31–1371 mg) in upper middle-income countries (*n* = 49), 134 mg/day (13–605 mg) in lower middle-income countries (*n* = 53), and only 47 mg/day (6–437 mg) in low-income countries (*n* = 28). At the extremes, the Maldives (upper middle-income) reported the highest estimated intake at 1409 mg/day, whereas Ethiopia (low-income) reported the lowest at 7 mg/day. Disparities were also evident when comparing world regions. In Sub-Saharan Africa and in Southern, Western, and Central Asia, estimated intakes were frequently below 100 mg/day. A similar pattern of variability was observed in Latin America and the Caribbean, where the regional median was 134 mg/day (range: 28–561 mg). Peru reported the highest intake in the region (208.9 mg/day), while Guatemala and Paraguay showed some of the lowest values (28.4 and 37.7 mg/day, respectively). Countries such as Mexico (130.9 mg/day), Brazil (104.3 mg/day), and Chile (141.1 mg/day) were above 100 mg/day, although still below international recommendations.

**Figure 1:**
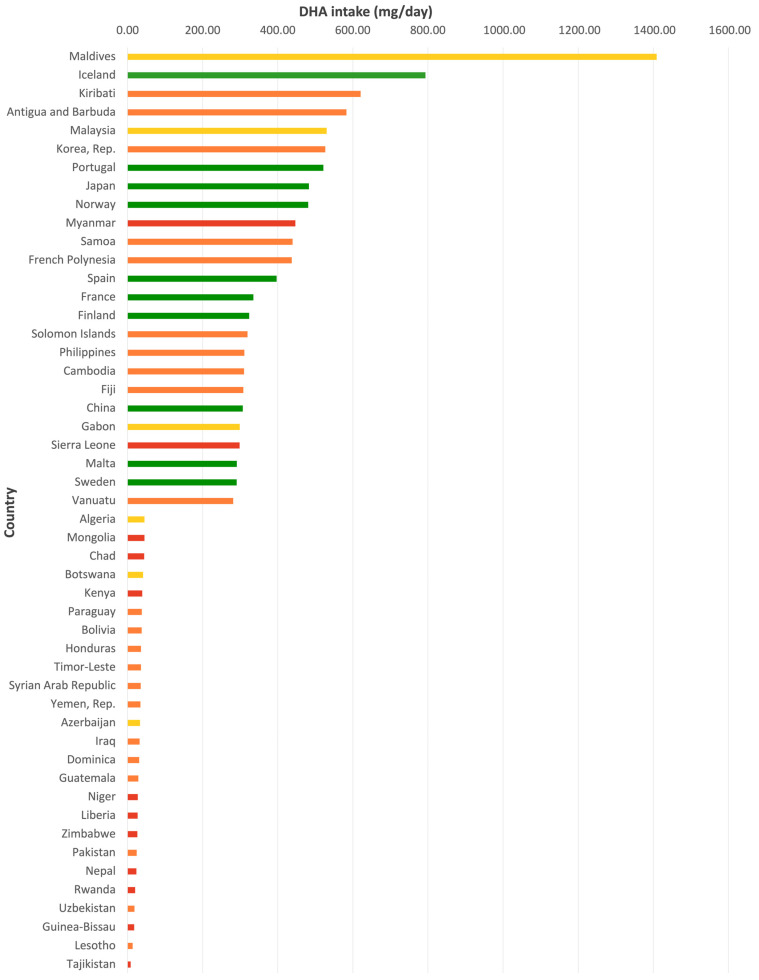
Top and bottom 25 countries by DHA intake (mg/day), color-coded by GNI classification. Top and bottom 25 countries by DHA intake (mg/day), color-coded by GNI classification. This figure presents the 25 countries with the highest and lowest estimated DHA intake based on FAO FBS data from Forsyth et al. [86]. Countries are color-coded according to the fiscal-year GNI classification used in the original dataset (red: low income; orange: lower-middle-income; yellow: upper-middle-income; green: high income). The visualization illustrates the marked variability in DHA intake across income groups, with high-income countries generally concentrated among the highest values and low-income countries among the lowest. The Maldives appears as an atypical case within the upper-middle-income category, consistent with reports documenting some of the highest per capita fish consumption worldwide in the Maldives, driven by tuna-centered dietary patterns [91]. GNI categories correspond to the fiscal-year classification applied in Forsyth et al. [86] and may differ from current World Bank definitions.

**Figure 2:**
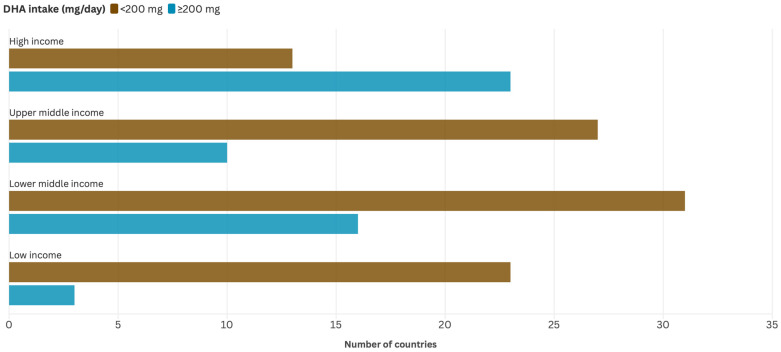
Number of countries with mean DHA intake below or above 200 mg/day, stratified by GNI classification. This figure is based on the global dataset reported by Forsyth et al. [86] and reflects country-level counts, where each country contributes equally regardless of population size. The 200 mg/day threshold is used as a reference value derived from expert consultation reports for early-life nutrition [12]. The figure illustrates that most low- and lower-middle-income countries fall below this threshold, whereas a greater proportion of high-income countries meet or exceed it, highlighting income-related disparities in DHA availability across countries. GNI categories correspond to the fiscal-year classification applied in Forsyth et al. [86] and may differ from current World Bank definitions.

**Figure 3:**
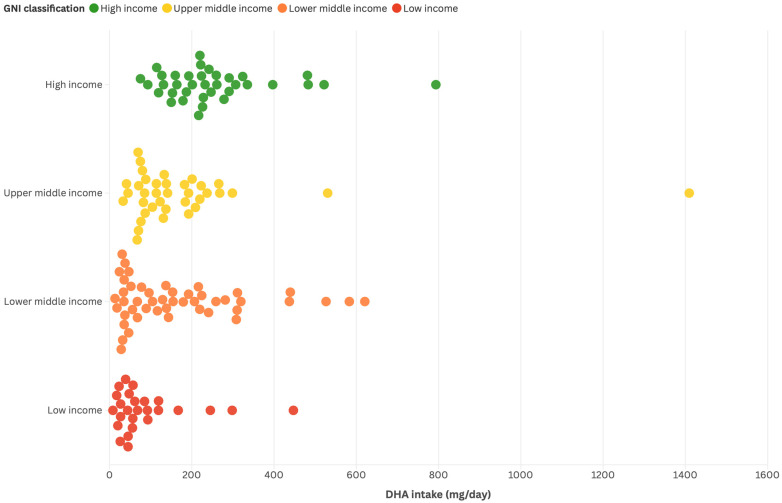
Distribution of DHA intake (mg/day) across countries, stratified by GNI classification. This figure, derived from the global dataset from Forsyth et al. [86], where each dot represents a country, illustrates the variability in DHA intake within and across income groups. High-income countries generally cluster at higher intake levels, while upper-middle-income and lower-middle-income groups display broader dispersion. Low-income countries are concentrated at the lower end of the distribution. These estimates are derived from FAO FBS data and reflect national-level DHA availability rather than individual consumption. GNI categories correspond to the fiscal-year classification applied in Forsyth et al. [86] and may differ from current World Bank definitions.

Furthermore, a comprehensive literature review by Cetin et al. [69] reports that 64% of countries (47 HICs and 128 LMICs) have average DHA intakes below 200 mg/day, with the lowest intakes found in sub-Saharan Africa and Central and South Asia. Together, these findings indicate a consistent relationship between gross national income and DHA intake, while also reflecting the modifying influence of geography and food systems. This pattern points to clear regional disparities in DHA consumption, with LMICs consistently reporting lower intake levels than HICs. To further illustrate these disparities in DHA intake across income groups and regions, three visualizations derived from Forsyth et al. [86] are presented: the 25 countries with the highest and lowest intakes (Figure 1), the proportion of countries above or below 200 mg/day by income classification (Figure 2), and the distribution of intakes across income classification (Figure 3).

In contrast with these findings derived from FAO FBS, we identified only one study with nationally representative data. Results from Landa-Gómez et al. [14], based on the 2012 and 2016 Mexican National Health and Nutrition Surveys (ENSANUT, for its acronym in Spanish), indicated a severe inadequacy in DHA + EPA intake among adults, with 99.6% of the population below the recommended intake levels in 2016, representing an increase from 96.3% in 2012 (*p* < 0.05). While Forsyth et al. [86] estimated DHA intake from global food supply data, Landa-Gómez et al. used individual dietary recall data, providing a more direct assessment of intake levels. Although DHA and EPA were not reported separately, the consistently high prevalence of inadequacy suggests that actual DHA intake may be even lower than global supply-based estimates.

While these findings are relevant, it is important to acknowledge the methodological limitations of estimating DHA intake using FBS as a proxy. These estimates are based on national-level data rather than using individual intake assessment instruments such as food frequency questionnaires or 24 h recalls [89]. While FBS offers useful data on macro-level food consumption patterns, it ignores differences in access to DHA-rich foods, variability within countries, and individual dietary habits [90]. Furthermore, there is no globally standardized technique for collecting DHA intake data, because not all countries conduct nationally representative dietary surveys to accurately estimate DHA intake. This highlights the need for more systematic methods to assess DHA consumption trends worldwide. These findings reinforce the urgent need for public health strategies to improve DHA intake in LMICs, addressing dietary accessibility and consumption patterns to reduce nutritional disparities across nations.

### 3.2. DHA Intake in Children

#### Patterns of DHA by Country Income Level

Evidence from cross-sectional studies across the world reflects an inadequate intake of DHA in school-aged children, showing variations according to the country’s income level, dietary patterns, and some associated metabolic factors. In high-income countries, such as Canada, lower red blood cell DHA levels were found in children with obesity, with a positive correlation between dietary DHA intake and blood status (r = 0.37, *p* = 0.003) [31], suggesting that although DHA availability in the blood may be higher, altered lipid metabolism in obesity could impact its bioavailability. In contrast, in LMICs, deficient DHA levels seem to be more linked to dietary availability and not so much to metabolic processes. In India, despite the high fish consumption observed in the child population, DHA intake remains insufficient, averaging 35.59 mg/day, with a moderate correlation (r = 0.376, *p* < 0.001) with plasma DHA levels [33]. Meanwhile, in China, plasma DHA levels were associated with diet quality, showing higher levels in children with a varied dietary pattern and lower levels in those whose diet was rich in snacks and sugary drinks [25]. In Zimbabwe, DHA levels in dried blood were lower than those reported in European children [26], while in Myanmar, in a food modeling study under different food availability scenarios, DHA remained a problem nutrient, reaching only 26% of the Reference Nutrient Intake (RNI) even in optimized diets. In this analysis, carp fish and eel were the only foods providing meaningful amounts of preformed DHA, while other items identified in the optimized combinations contributed to overall dietary adequacy but not to DHA specifically [32]. These findings highlight the urgent need for intervention strategies aimed at improving DHA intake in vulnerable populations. Summary of evidence on dietary DHA intake in children: differences by national income level are shown in Table 1. This distribution reflects the limited availability of DHA intake data from low- and middle-income countries and highlights the uneven global evidence base for estimating dietary DHA intake across settings.

**Table 1:** Summary of evidence on DHA intake, dietary exposures, and biomarkers across studies, stratified by national income level.

Author, Year, Country	GNI	Domain	Study Design	Life Stage	DHA Assessment	Reported Diet and/or Biomarkers	Main Outcome
Yuan, 2022 [72] France	High income	Dietary patterns and biomarkers	Cohort study (EDEN mother–child cohort, *n* ≈ 2000)	Children (perinatal: maternal pregnancy)	Biomarkers (maternal RBC membrane, cord RBC membrane, colostrum)	Patterns of dairy product consumption during pregnancy (derived from FFQ + PCA): Cheese Reduced-fat dairy products Semi-skimmed milk and yogurt	Maternal RBC membrane: Cheese β = −0.01 (95% CI: −0.05, 0.07); Reduced-fat DP β = 0.10 (95% CI: 0.06, 0.15); Semi-skimmed milk/yogurt β = 0.10 (95% CI: 0.05, 0.14). Cord blood RBC membrane: Cheese β = 0.02 (95% CI: −0.04, 0.09); Reduced-fat DP β = 0.06 (95% CI: 0.02, 0.11); Semi-skimmed milk/yogurt β = 0.05 (95% CI: 0.00, 0.10). Colostrum: Cheese β = −0.02 (95% CI: −0.09, 0.05); Reduced-fat DP β = 0.03 (95% CI: 0.02, 0.09); Semi-skimmed milk/yogurt β = 0.04 (95% CI: −0.02, 0.09). Note: Associations reflect dietary pattern correlations with DHA status and should not be interpreted as direct evidence of DHA content in dairy foods. * Adjusted for study center, maternal age, sampling day (colostrum), maternal healthy dietary pattern, and fish consumption; sensitivity analyses excluded gestational diabetes, hypertensive disorders, extreme energy intake, and preterm delivery.
Mulder, 2022 Canada [45]	High income	Neurodevelopment	Cross-sectional study (with follow-up subgroup from a previous RCT)	Children, 5–6 years (mean 5.75 y)	Dietary assessment: FFQ (*n* = 280), 1 × 24 h recall (*n* = 272), 3 × 24 h recalls (*n* = 259) Biomarker: RBC fatty acids (*n* = 245), measured by gas–liquid chromatography.	DHA intake (mg/day) RBC DHA (% total fatty acids):	Median DHA intake: 52.9 mg/day (FFQ) and 19.5 mg/day (24 h recall; *p* < 0.001). RBC DHA and cognitive outcomes: Higher RBC DHA was associated with better short-term memory (KABC Sequential, rho = 0.187, *p* = 0.019) and vocabulary (PPVT, rho = 0.211, *p* = 0.009). Q5 vs. Q1 RBC DHA memory scores: 5.80% ± 1.51% vs. 4.93% ± 1.34%, *p* = 0.012. Dietary DHA correlations: Dietary DHA correlated with memory (rho = 0.221, *p* = 0.003). Ethnic differences: RBC DHA was higher in Chinese (6.06% ± 1.42%) vs. White children (5.38% ± 1.52%), *p* = 0.013. * Adjusted for ethnicity and relevant child/family characteristics (e.g., parental IQ, household composition); analyses restricted to White children in some models.
Huang, 2022 China [25]	Upper-middle-income	Intake	Cross-sectional study	Children (5–7 years)	Plasma and erythrocytes (Gas Chromatography–Mass Spectrometry)	Diversified pattern: high intakes of fruits, nuts, leafless vegetables, poultry, fungi and algae, fresh beans, tubers, fish, meat, soybeans and products, snacks, rice, shrimp, crab, shellfish Plant pattern: coarse cereals, soybeans and products, leafless vegetables, tubers; low poultry and meat Beverage/snack pattern: high beverages, snacks, milk and products; low shrimp, crab, shellfish, fish	Median DHA levels: Plasma 7.91 µg/mL (IQR 6.22–10.45), RBC 13.89 µg/mL (IQR 7.49–18.99). Diversified dietary pattern: positively associated with plasma DHA (β = 0.145, 95% CI: 0.045–0.244, *p* = 0.004); correlations with eggs, meat, poultry, and fish. Beverage/snack pattern: weak negative association with plasma DHA (β = −0.092, 95% CI: −0.187–0.003, ~*p* = 0.05). Risk patterns: plasma DHA inversely related to obesity risk pattern (OR = 0.873, 95% CI: 0.786–0.969, *p* = 0.011) but positively associated with blood lipid risk pattern (OR = 1.271, 95% CI: 1.142–1.415, *p* < 0.001). RBC DHA: limited associations; only significant with blood lipid risk pattern: (OR = 1.043, 95% CI: 1.002–1.086, *p* = 0.040) * Adjusted for age, sex, caregiver, caregiver’s education and occupation, family economic level; additional adjustment for meat, poultry, eggs, and fish intake in some model
Forsyth, 2016 [86] Multi-country (175 nations, global analysis).	175 countries, grouped by World Bank classification: high, upper-middle, lower-middle, and low income.	Intake (national per capita DHA availability)	Ecological study using FAO FBS (2009–2011), fatty-acid composition from NUTTAB 2010, adjusted for retail/household wastage	Not applicable (national-level ecological estimates)	Estimated per capita DHA availability from FAO FBS combined with food fatty-acid composition tables (NUTTAB 2010)	Dietary availability only (no individual dietary surveys; no biomarkers)	Global DHA intake (by GNI): High-income: 192 mg/day (range: 67–706; *n* = 42) Upper-middle-income: 122 mg/day (range: 31–371; *n* = 49) Lower-middle-income: 134 mg/day (range: 13–605; *n* = 53) Low-income: 47 mg/day (range: 6–437; *n* = 28) Extremes: Highest intake: Maldives (1409.3 mg/day) Lowest intake: Ethiopia (7.01 mg/day) Sub-Saharan Africa: several countries with intakes < 50 mg/day; Ethiopia lowest (7.0 mg/day). Southern, Western, and Central Asia: many countries within 20–60 mg/day, reflecting very limited DHA intake. Small island states: highest median intake, 204.7 mg/day, consistent with reliance on marine foods. Latin America: median 134.2 mg/day (range: 28–561). Peru: 208.9 mg/day (highest); Guatemala: 28.4 mg/day; Paraguay: 37.7 mg/day (lowest) Sources and modifiers: Fish and seafood: main contributors to DHA supply worldwide. Landlocked countries: substantially lower intake (median 47.4 mg/day). Correlations: National birth rates negatively associated with DHA intake (r = −0.277; *p* < 0.001). * No individual adjustment; ecological estimates based on FAO FBS, corrected for wastage; stratified by GNI, geography (coastal vs. landlocked), and birth rates.
Mak, 2020 Canada [31]	High income	General development	Cross-sectional analysis (baseline data of an intervention trial)	Children with obesity (*n* = 63, 6–13 years, Tanner stages 1–3)	Dietary intake (3-day food diaries) and RBC fatty acids (gas chromatography)	Fish/seafood intake (servings/day) Dietary EPA, DHA, and EPA + DHA (mg/day) RBC EPA, DHA, and EPA + DHA (% of total fatty acids)	Adiposity and DHA: Higher body fat percentage was associated with lower RBC DHA (Tertile 1: 2.45% ± 0.84% vs. Tertile 3: 1.86% ± 0.35%; *p* < 0.05) and lower RBC EPA + DHA (Tertile 1: 2.24% ± 0.86% vs. Tertile 3: 1.86% ± 0.35%; *p* < 0.05). Diet–biomarker correlations: Dietary DHA correlated positively with RBC DHA (r = 0.37; *p* = 0.003). Each additional serving of fish increased RBC EPA + DHA by 1.1% (*p* = 0.0005). Dietary patterns: Children with higher adiposity consumed less fish (0.2 ± 0.04 servings/day) and had lower fruit/vegetable intake (*p* = 0.02). * Adjusted for age, sex, Tanner stage, race, and DXA-measured body fat; analyses stratified by sex and adiposity tertiles.
Soe, 2020 Myanmar [32]	Upper-middle-income	General development	Cross sectional study	Children (primary school)	Dietary intake modeling (24 h recall, 5-day food record, weighed records, nutrient composition tables from Vietnam and USDA)	Reported food groups tested in Optifood modeling Fish (carp, eel) identified as only foods contributing meaningful preformed DHA Other foods (shrimp, duck eggs, water spinach, peas) included in nutrient-dense combinations, but not as DHA sources	Nutrient-dense food combinations: Optimized diets included carp fish (7×/week), eel, shrimp (5×/week), duck eggs (3×/week), water spinach (4×/week), peas (4×/week). DHA adequacy: Even with optimized diets, DHA intake reached only ~26% of the RNI. Note: Carp fish and eel were the only direct dietary sources of DHA; other foods in the modeled combinations contributed to nutrient adequacy but not to DHA. * No individual adjustment; linear programming model using Optifood with constraints for food availability, affordability, cultural patterns, and nutrient composition.
Ogaz-Gonzalez, 2018 [75] Mexico	Upper-middle-income	Neurodevelopment	Cohort study (*n* = 142 mother–child pairs)	Pregnancy/Children (42–60 months)	Dietary intake (FFQ, 1st and 3rd trimester); Interaction analyses with maternal serum DDE	Reported diet DHA intake	Maternal intake: 35.1 mg/day (1st trimester) and 31.5 mg/day (3rd trimester). Effect modification: Low maternal DHA intake amplified the negative association between prenatal DDE exposure and motor development. Associations: Low-DHA group: β = −1.25 (95% CI: −2.62, −0.12). High-DHA group: β = 0.50 (95% CI: −0.55, 1.56). Sex differences: Protective effect of higher DHA intake was particularly observed in girls. Adjusted for child’s age at exam, HOME score, sex, maternal IQ, breastfeeding, energy intake, and maternal DDE levels; sensitivity analyses adjusted DHA/ARA for other PUFAs and stratified by sex.
Hakola, 2017 [76] Finland	High income	General development	Prospective cohort study (DIPP birth cohort, *n* ≈ 3807)	Pregnancy (maternal diet during late pregnancy), offspring childhood	Maternal dietary intake estimated with FFQ; nutrient composition from Finnish Food Composition Database	Reported dietary DHA intake (from fish and other food sources)	Maternal DHA intake (late pregnancy): Mean intake was 123 mg/day in children with obesity vs. 126 mg/day in children without obesity; no association with childhood obesity. Boys: Maternal DHA intake was 124 mg/day in boys with obesity vs. 125 mg/day in boys without obesity; no association observed. Girls: Maternal DHA intake was 122 mg/day in girls with obesity vs. 127 mg/day in girls without obesity; no association observed. * Adjusted for maternal early pregnancy BMI, gestational weight gain, timing of first weight measurement, glucose intolerance diet, education, smoking during pregnancy, and breastfeeding duration; analyses stratified by sex.
Gershuni, 2021 [81] USA	High income	General development	Prospective cohort with embedded case–control study (cases = 16 women with SPTB; controls = 32 women with term delivery, matched by race and obesity)	Pregnancy (second trimester, 20–26 weeks gestation)	Dietary intake (three 24 h recalls) Fecal and plasma metabolomics	Dietary exposures (3 × 24 h recalls, NDSR): total ω-3 fatty acids, DHA, EPA, saturated fat (palmitate), fiber, total energy intake. Supplements: all participants reported prenatal vitamin use (DHA content not captured in recalls). Biomarkers: fecal untargeted metabolomics (including DHA, EPA); plasma untargeted metabolomics.	Dietary intake: Women with SPTB had slightly higher DHA intake than controls, but levels remained very low in both groups (0.18 ± 0.34 g/day vs. 0.11 ± 0.25 g/day; *p* = 0.370). Total ω-3 intake was higher in SPTB cases (2.65 ± 1.05 vs. 1.89 ± 0.89 g/day; *p* = 0.014). Saturated fat intake was also higher in SPTB (31.38 ± 7.37 vs. 26.08 ± 8.62 g/day; *p* = 0.045). Fecal biomarkers: Women with SPTB showed higher fecal DHA and EPA levels (FDR < 0.20) despite low reported intake (<0.2 g/day). Plasma biomarkers: Elevated DHA-derived plasma metabolites were identified in SPTB cases, suggesting alterations in fatty acid metabolism. Correlations: Fecal DHA/EPA were positively correlated with saturated fat intake (*p* < 0.05) Matched for race/ethnicity and prepregnancy BMI; controls excluded if pregnancy complications; dietary data cleaned for implausible energy intake.
Hautero, 2017 [38] Finland	High income	Intake	Cross-sectional study (mothers in late pregnancy and infants at 1 month)	Pregnancy and lactation/early infancy	Biomarkers (serum phospholipids DHA in mothers and infants, GC analysis)	Fish intake frequency (0, 1, 2, 3, ≥4 times/week) Healthy Eating Index score (tertiles) 3-day food diaries and FFQ for dietary intake	Maternal serum phospholipid DHA: Frequent maternal fish intake (≥3×/week or ≥36 g/day) significantly increased DHA levels compared with low intake (*p* < 0.001). Infant serum phospholipid DHA: Infants of mothers with higher fish intake also showed significantly higher DHA levels at 1 month of age (*p* < 0.001). Correlation: Maternal and infant DHA were strongly correlated (R = 0.582, *p* < 0.001). * Adjusted for maternal age, pre-pregnancy BMI, parity, and education; GEE used for persistent fish intake and diet quality analyses.

GNI, Gross National Income; DHA, Docosahexaenoic Acid; EPA, Eicosapentaenoic Acid; ARA, Arachidonic Acid; PUFAs, Polyunsaturated Fatty Acids; LCPUFAs, Long-Chain Polyunsaturated Fatty Acids; ALA, Alpha-Linolenic Acid; LA, Linoleic Acid; SPTB, Spontaneous Preterm Birth; FFQ, Food Frequency Questionnaire; RCT, Randomized Controlled Trial; PCA, Principal Component Analysis; DP, Dietary Patterns; RBC, Red Blood Cell; DDE, 1,1-dichloro-2,2-bis (*p*-chlorophenyl) ethylene; MSCA, McCarthy Scales of Children’s Abilities; KABC, Kaufman Assessment Battery for Children; PPVT, Peabody Picture Vocabulary Test; IQ, Intelligence Quotient; IQR, Interquartile Range; FAO, Food and Agriculture Organization; FBS, Food Balance Sheets; DXA, Dual-Energy X-ray Absorptiometry; USDA, United States Department of Agriculture; RNI, Reference Nutrient Intake; HOME, Home Observation for Measurement of the Environment; NDSR, Nutrition Data System for Research; FDR, False Discovery Rate; GC, Gas Chromatography; GEE, Generalized Estimating Equations; OR, Odds Ratio; CI, Confidence Interval; BMI, Body Mass Index; HICs, High-Income Countries; LMICs, Low- and Middle-Income Countries; ENSANUT, Mexican National Health and Nutrition Survey; MeSH, Medical Subject Headings; MetS, Metabolic Syndrome. GNI categories follow the World Bank Atlas method, fiscal year 2023, based on 2022 GNI per capita. Relevant outcomes related to DHA in different health domains (e.g., general development, neurodevelopment, immune system, etc.) are presented. All included studies directly or indirectly assessed DHA exposure through dietary reports, biochemical biomarkers, or both. Reported dietary exposures indicate food groups or nutrients as described in each original study. * Indicates confounders for which the analyses were adjusted, when available, as reported in the Main outcome section of each study.

### 3.3. The Role of DHA in Child Health

#### 3.3.1. General Development and DHA Dietary Intake

General development in children involves the establishment and maturation of key physiological organ systems, including but not limited to the following: gross motor skills, physical growth, cardiovascular system and function, lung maturation, and body composition. Accordingly, Mitguard et al. [18]. conducted a systematic review of the literature published between 2005 and 2020, including 21 studies that found that increased DHA levels in breast milk during the first three months postpartum were associated with enhanced motor development at 6.6 months of age (*p* < 0.005). Similarly, higher DHA content in milk at 3 days postpartum was positively associated with cognitive scores on the Griffiths Mental Development Scale at 2.5 years, specifically, hand–eye coordination, performance, and language. Another literature review reported a prospective study where higher DHA concentrations during infancy were associated with better-sustained attention and psychomotor development at 5 years of age [67].

Regarding physical growth and body composition, higher breastfeeding DHA content at 6 weeks postpartum was associated with a higher ponderal index at 12 months (r = 0.15) and higher lean body mass at 2 years, with the effect still persistent until at least 5 years of age (*p* < 0.05) [18]. Similarly, Delplanque et al. [67] in 2015 reported evidence in a literature review from an observational study with 500 infants fed with breastmilk high in DHA or formula-fed infants; children who were breastfed had higher weight gain and circumference growth vs. formula-fed infants.

In accordance with the aforementioned, observational studies also confirmed these results, demonstrating direct associations between DHA levels and child development outcomes. A cohort study conducted with 39 children and their mothers in Poland with a follow-up of 2 years found a DHA content of 0.50% of total fatty acids in breast milk, corresponding to a better performance on the motor skills subscale of the Infant Development Scale at the age of 6 months (β = 0.275, R^2^ = 0.38, *p* ≤ 0.05) [71]. In another cohort study of mothers in the Netherlands, maternal plasma LCPUFAs concentration during pregnancy was assessed in relation to blood pressure in children at 6 years of age, which found significantly lower systolic blood pressure with higher levels of DHA (reduction: 0.29 mmHg; 95% Confidence Interval [95% CI]: −0.54, −0.03; *p* < 0.005), indicating a potential longer-term cardiovascular benefit [30].

#### 3.3.2. General Development and DHA Supplementation

Diverse systematic reviews, narrative reviews, and randomized clinical trials have tested the effects of DHA on various general development outcomes, frequently in the context of high-income countries, all of which reported contrasting results depending on the targeted population group, the dose, and the genetic factors involved. DHA and arachidonic acid (ARA) supplementation in full-term infants were associated with improved visual acuity at 12 months in a literature review of clinical trials and observational studies around the world, and DHA supplementation in premature infants was associated with a 0.5 cm larger head circumference at 6 months of corrected age in the United States, potentially indicating a benefit in brain growth [8].

Another review of the literature reported a clinical trial that showed that supplementing with 0.3% DHA in the diet resulted in a 25% reduced incidence of bronchopulmonary dysplasia in a clinical trial of 1000 term and preterm infants in Australia, while preterm infants fed higher doses had an increment of 0.7 cm in height in comparison to standard feeding practices at 18 months of corrected age; furthermore, a higher intake of DHA during the first months of life has been documented in relation to visual development in premature infants, with greater visual acuity observed compared to individuals with a lower intake of this important LCPUFA [67]. These findings support the role of DHA in the maturation of essential organs and systems in early life, although health status at birth may gate this impact.

Concerning evidence from studies in LMICs, the results are less consistent in terms of lung function and metabolic health. A randomized clinical trial was published in 2017 in Mexico, comprising 772 children followed from birth to at least 5 years of age, which observed no significant differences in lung function parameters between the two DHA and placebo groups, nor in their trajectory of lung development between 3 and 5 years of age [51].

In contrast, in an analysis of the infant metabolome at 3 months, prenatal DHA supplementation altered important metabolic pathways, including amino acid and aminoglycan metabolism in children of mothers carrying the minor allele Homozygous genotype for the T allele (TT)/Total Cholesterol (TC) of the Fatty Acid Desaturase 2 (FADS2) gene, whereas carriers of the Carbon Copy/Homozygous Genotype (context-dependent) (CC) showed an increase in tricarboxylic acid (TCA) cycle metabolites and enhancement of galactose metabolism [54]. These results indicate that children’s metabolic response to DHA supplementation may be modulated by the maternal genetic profile, emphasizing the importance of genetic analysis in supplementation approaches. In another study conducted in Mexico, children’s cardiometabolic health was assessed at 11 years of age, and it was found that, in children of mothers with the TT genotype in FADS2, DHA supplementation decreased the metabolic syndrome score by 0.26 units, whereas in children of mothers with the CC genotype, the metabolic syndrome score increased by 0.24 units [52]. This indicates that the effect of DHA supplementation on metabolic regulation in children may be modulated by the genetics of mothers.

#### 3.3.3. Neurodevelopment and DHA Dietary Intake

Three comprehensive literature reviews [3,6,67] explain the essential role of DHA in neurodevelopment during childhood: in a 2014 publication including systematic reviews, Mallick et al. [3] found that approximately 90% of all *n*-3 PUFAs in brain tissue consist of DHA, which is primarily found in gray matter and synaptic membranes, and pointed out the possible involvement of this fatty acid in synaptic plasticity and neurotransmission. Diets enriched with DHA and other *n*-3 PUFAs have been reported to improve performance in verbal learning, spelling, and reading [6], as well as to improve executive function and reduce oppositional behavior and hyperactivity in children. Furthermore, DHA has also been reported to positively influence the endocannabinoid system, an important modulator of mood, pain, and cognition [67].

Likewise, two literature reviews [4,6], including observational studies and RCT, primarily from high-income countries, found further evidence of an association between DHA and cognitive development in school-aged children; in one study, including a group of 1200 children (aged 7–9 years), it was shown that a six-month diet rich in *n*-3 PUFAS increased plasma and erythrocyte DHA levels, resulting in improved language skills, memory, and academic performance. Likewise, Basak et al. [4] found a 0.8-to-1.8-point increase in childhood IQ associated with a higher maternal DHA intake, although the effects were inconsistent for academic and cognitive performance at older ages in the RCTs contributing to that review.

Similar results were obtained in individual studies as well; a clinical trial with 205 German children (4–6 years old) showed that children eating Atlantic salmon three times weekly for 16 weeks had significantly higher plasma and buccal cell DHA levels than the meat-eating group [62]. Salmon consumption correlated with higher plasma DHA (Spearman’s rho = 0.302, *p* = 0.003), and there were associatively specific improvements in two domain cognitive tasks: symbol search (*p* = 0.038) and concept in pictures (*p* = 0.047) with no overall group IQ difference [62]. In addition, two observational studies in high-income countries reported similar findings. A cohort study of 166 children of mothers with obesity in Denmark found that DHA status in erythrocytes at 9 months of age was associated with child development at 3 years and that polymorphisms in the FADS influence this relationship. The study showed higher median Red Blood Cells (RBC DHA) levels connected with greater personal–social skills (β = 1.8, 95% CI: 0.3, 3.3; *p* = 0.019) and problem-solving ability (β = 3.4, 95% CI: 1.2, 5.6; *p* = 0.003). In addition, the authors found that polymorphisms in FADS genes (rs1535 and rs174575) were associated with higher measures of social development (β = 2.6, 95% CI: 0.01, 5.1; *p* = 0.011) [2]. This indicates that the relationship between DHA and cognitive and social development may be influenced by genetic factors.

In addition, a cross-sectional study in Canada including 5- to 6-year-old children found increased levels of RBC of DHA positively associated with higher scores of memory and language as measured with the Peabody Picture Vocabulary Test (PPVT; rho = 0.211; *p* = 0.009), as well as on the Kaufman Assessment Battery for Children (KABC) sequential scale, which serves as a measure of short-term memory [45] (rho = 0.187; *p* = 0.019). In addition, RBC-DHA levels differed based on the ethnicities of the children participating, where higher levels were found in the Chinese descent group compared with the White descent group (6.06% ± 1.42% vs. 5.38% ± 1.52%, *p* = 0.013), suggesting dietary and genetic effects on the availability and effects of DHA upon cognitive development.

Furthermore, possible genetic interactions influencing DHA availability have been pointed out, indicating a polymorphism that could modulate the response to DHA; one study evaluated children with low LCPUFA synthesis genotypes in the FADS gene and reported that children whose diets had a higher DHA content, compared to younger children, showed four-point-higher IQ scores at 8 years of age [67]. This highlights that, despite the presence of genetic variants that may affect DHA synthesis, adequate dietary DHA intake is critical to maintaining the necessary DHA levels to enhance child neurodevelopment.

#### 3.3.4. Neurodevelopment and DHA Supplementation

We identified one systematic review [85] and seven narrative reviews [3,6,8,20,22,65,70] on supplementation and neurodevelopment, all of them from high socioeconomic-income countries that are consistent with the findings outlined above. In infants, intake of DHA-enriched complementary foods can convey improvement in visual acuity at both 9 and 12 months of life, which may result in a more developed visual cortex [85]. At school age, DHA supplementation benefits learning and attention: the effects are higher among those children who have previously under-performed. From two narrative reviews, DHA improves reading, spelling, and verbal memory, with its impact being greater in children who start from a lower level of ability [22,65].

In addition, the literature analysis by Cardoso et al. [20] reported results from clinical trials, with an RCT finding that 600 mg/day of DHA for four months improved academic performance in children with learning problems, and that a dose of 240 mg/day of DHA resulted in clinical and biochemical improvement in 66% of children with autism. Similarly, 1032 mg/day of DHA was administered to children with attention deficit/hyperactivity disorder (ADHD), resulting in improved attention, behavior, and literacy, with no consistent findings on hyperactivity [6]. There is evidence that supplementing DHA to premature newborns may have a positive impact on their early neurocognitive development; higher doses of DHA (0.86–1% of total PUFAs) in premature newborns have been related to improved solving and memory skills at 6 months of age, as well as a reduction in symptoms of autism and ADHD [70]. A review by Andersen [2] of the literature reflected evidence from observational studies, showing that higher DHA availability in erythrocytes is associated with improved personal–social development and problem-solving, with a variation influenced by various polymorphisms in the FADS gene.

#### 3.3.5. Immune System Modulation and DHA

Two literature reviews from high-income countries [6,7] examined the modulatory role of DHA on the infant immune system, with impacts observed in regulating inflammation and modulating the adaptive immune response. According to Weiser et al. [6], metabolites of DHA, including resolvins, protectins, and maresins, are important in the resolution of inflammatory processes and reductions in chronic responses observed in autoimmune diseases and neuroinflammation. Moreover, DHA has been shown to diminish microglial activation and the production of pro-inflammatory cytokines (e.g., IL-1β and IL-6) in an animal model of inflammatory disease of the nervous system, and DHA supplementation promoted a response that included the regulation of pro-inflammatory cytokines (IL-6 and TNF-α) by 30% in randomized clinical trials. In another review [7], it was indicated that DHA decreases the production of pro-inflammatory cytokines derived from ARA, IL-1, IL-2, IL-6, IFN-γ, and TNF-α, providing greater stability in the immune balance and, consequently, a lower risk of chronic inflammation. These observations confirm that DHA functions as a major coordinate in the infant immunity system, with direct efficacy in lowering systemic inflammatory response and neuroinflammation, which has important consequences for preventing and managing inflammatory disorders in early life stages.

### 3.4. Maternal DHA Intake and Pregnancy Outcomes

#### 3.4.1. Patterns of DHA Intake During Pregnancy and Its Fetal Implications

Maternal DHA intake during pregnancy varies depending on dietary habits, access to food sources, and socioeconomic status. In this review, we found mostly high socioeconomic income studies addressing the effects of DHA intake during pregnancy and its maternal and child effects. Burdge et al.’s [92] narrative review identified observational studies that documented lower DHA dietary intakes in vegetarian women than omnivores, with plasma levels being 20–47% lower and dietary intakes of 10–17 mg/day vs. 150–316 mg/day for omnivores. This deficiency was also seen in neonates of vegetarian mothers, with 32% less DHA in umbilical cord plasma and 69% less DHA in RBC in vegan mothers. This finding was corroborated in single studies, such as the one conducted by Crozier, which showed that vegetarian women in the United Kingdom had significantly lower levels of DHA in their serum phosphatidylcholine (57.8 vs. 82.6 µg/mL, *p* < 0.001) [80].

In individual studies conducted in high-income countries like Japan, maternal food intake of fish and seafood can lead to an increased DHA intake of 500–600 mg/day for pregnant women and is associated with higher DHA levels in breast milk and erythrocytes. A clinical trial in the United Kingdom reported that salmon consumption from week 20 to 38 of gestation increased DHA levels in maternal erythrocytes from 4.33% to 5.02%, and in neonates by up to 6.10% at birth, and a positive correlation was observed between maternal and neonatal DHA levels (r = 0.384, *p* = 0.030) [53], indicating a maternal–fetal transfer favored by the consumption of fatty fish.

On the other hand, middle-income countries like Chile are characterized by a high intake of *n*-6 fatty acids and a low intake of DHA in the maternal diet. Bascuñán et al. [35] found a level of 3.6 ± 0.6% of DHA as the predominant *n*-3 PUFAs in the erythrocytes and membrane phospholipids of 80 Chilean pregnant women; however, the average intake was 40 mg/day, well below the FAO/WHO recommendation of 200 mg. These data indicate an imbalance in the *n*-6/*n*-3 ratio and a high proportion of *n*-6 fatty acids in the diet, which may restrict the endogenous conversion of ALA to DHA. Therefore, viable approaches to promoting enhanced DHA intake among pregnant women, either as dietary or supplemental sources, need to consider the optimal fetal transfer of this important fatty acid.

#### 3.4.2. DHA and Its Association with Fetal Growth and General Development

Maternal intake of DHA has been extensively examined with respect to fetal development and pregnancy outcomes. The review by Cetin et al. [69], primarily compiling findings from high-income countries, concluded with strong evidence for the role of DHA in preterm birth (PTB) prevention. The authors highlighted that Cochrane’s meta-analysis of 70 randomized clinical trials (19,927 women) demonstrated that DHA supplementation lowered the risk of early PTB (<34 weeks) by 11% (RR = 0.89; 95% CI: 0.81, 0.97) and early PTB by 42% (RR = 0.58; 95% CI: 0.44, 0.77). A later update, including 36 studies and 23,726 women, found reductions of 12% for PTB (RR = 0.88, 95% CI: 0.81, 0.95) and of 35% for early PTB (RR = 0.65, 95% CI: 0.46, 0.92). In the Assessment of DHA on Reducing Early Preterm Birth clinical trial (ADORE) (1100 pregnancies), supplementation with 1000 mg/day DHA reduced early PTB by 51.2% in women with low DHA status (<6% in RBC phospholipids) while in Omega-3 to Reduce the Incidence of Preterm Birth (trial) (ORIP) (5517 women) a significant reduction was seen with 800 mg DHA + 100 mg EPA, but these results were attributed to women with very low baseline levels. In a secondary analysis of the ADORE study, the risk of early PTB (3.45% vs. 1.2%) was reduced by 65% in women with <6% erythrocyte DHA [69]. These findings support the observation that the benefits of supplementation are more prominent in women with DHA deficiency and are pertinent to the prevention of adverse events linked to fetal growth and maturation.

In contrast, observational studies provide more heterogeneous findings. In a cohort from Mexico [77], it was reported that greater maternal DHA intake in the second trimester was associated with shorter birth length (β = −0.34 cm per 1 SD increase; 95% CI: −0.10, −0.59), and no changes in gestational age. In the long term, it was associated with a lower peripubertal height z-score (second trimester: β = −019, 95% CI: −034, −003; third trimester: β = −019, 95% CI: −033, −006) and with a lower BMI (Body Mass Index) z-score (β = −024, 95%CI: −046, −002), which was attenuated when adjusted for height (β = −016, 95%CI: −037, 006) [77]. With respect to evidence from high-income countries, Gershuni et al. [81], in a prospective case–control study of 48 subjects in the USA, found that women with spontaneous preterm birth (SPTB) showed higher fecal metabolome levels of DHA and EPA, even though they were taking 200 mg/day of DHA by supplement (False Discovery Rate < 2). Although both groups were characterized by low omega-3 intake (<0.2 g per day), there were no significant differences in dietary omega-3 intake (*p* = 0.370) and plasma DHA-derived metabolites (14-HDoHE/17-HDoHE) and omega-6/omega-3 ratio were significantly higher in the SPTB versus control cohort (8.25:1 SPTB; 9.14:1 controls); therefore, a proinflammatory/oxidative stress environment might have been induced in the SPTB cohort, which could also influence DHA metabolism. These findings emphasize the need to consider not only the amount of DHA ingested, but also the basal status, the balance with other PUFAs, and the metabolic context in which its utilization occurs, suggesting that a pro-inflammatory condition might correspond to diet type, which might, in turn, affect DHA metabolism and potentially pose a risk factor for preterm birth.

In terms of general development, maternal DHA supplementation has also been extensively investigated regarding its effects on maternal and child health factors other than gestational age. A systematic review from Mitguard et al. [18], which was based mainly on data from HICs, showed that maternal stores of DHA can become depleted in multiple pregnancies when intake is inadequate. Particularly, in a randomized, double-blind clinical trial in a middle-income countries, Ramakrishnan et al. [60] assessed the impact of prenatal supplementation with 400 mg per day DHA at 18 months of age in Mexican mothers and found no difference between the intervention and placebo groups in mental development scores (DHA: 94.3 ± 10.7 vs. placebo: 95.2 ± 9.3; adjusted difference = −1.00, 95% CI: −2.42, 0.42), psychomotor development (DHA: 93.0 ± 8.9 vs. placebo: 93.3 ± 9.8; adjusted difference = −0.46, 95% CI:−1.80, 0.88), or on the behavior scale (DHA: 111.5 ± 6.7 vs. placebo: 111.5 ± 6.2; 95% CI: −0.95, 0.93). An interaction was identified between the quality of the home environment and psychomotor development (*p* = 0.03), suggesting that DHA supplementation seems to mitigate the negative effects of low home stimulation. One possible implication of these findings is that there may be a more marked effect of maternal supplementation among populations who are at higher risk for poor DHA supply and are thus in greater need, such as mothers of multiples and children who have experienced low-stimulation environments.

#### 3.4.3. Metabolic and Epigenetic Effects of Prenatal DHA

Compelling data on the in-depth metabolic and epigenetic effects of prenatal DHA supplementation have emerged from RCTs in upper-middle-income countries like Mexico. Lee et al. [49] showed that the supplementation of 400 mg/day DHA from the 18th week of gestation until delivery altered the methylation of important imprinted genes related to fetal growth, including Insulin-like Growth Factor 2 (IGF2) and H19. While there were no significant group differences in a global analysis, preterm births were associated with greater IGF2 Promoter 3 (P3) (*p* = 0.04), offspring of overweight mothers had a greater IGF2 differentially methylated region (DMR) (*p* = 0.03), and offspring of mothers with a normal BMI had lower imprinted maternally expressed transcript (H19) DMR (*p* = 0.01), possibly in a DHA-dependent manner, suggesting that DHA modulates the epigenetic programming of the fetal environment.

Furthermore, genetic interactions have been examined in studies based on data from the same clinical trial. Tandon et al. [54] found that the three-month infant metabolome response to DHA differed according to the maternal FADS2 rs174602 genotype: DHA supplementation enriched the following amino acid and aminoglycan pathways in infants whose mothers were carriers of the minor (A) allele, whereas in mothers who were non-carriers, DHA reduced metabolites of the TCA cycle and galactose metabolism. That genotype–supplementation interaction was also at play when offspring were assessed at 11 years of age, where DHA supplementation corresponding to the TT maternal genotype was related to a lower metabolic syndrome score (Δ = −0.26; *p* = 0.09), but to a higher metabolic syndrome score in offspring when mothers were CC (genotype carriers (Δ = 0.24; *p* < 0.01), which emphasizes the requirement to incorporate maternal genetic profiles in future supplementation strategies.

In contrast, other clinical trials conducted in the same socioeconomic context have shown neutral results. In Mexico, Gutiérrez-Gómez et al. [61] assessed 524 children at 4 years of age following prenatal supplementation (400 mg/d of DHA) and found no significant differences in TC, High-Density Lipoprotein (HDL), Low-Density Lipoprotein (LDL), triglycerides, glucose, or insulin between the intervention and placebo groups. No differences were found in the prevalence of dyslipidemia or glycemic alterations, despite the presence of high adherence to the intervention, with demonstrated increases in DHA levels in umbilical cord blood and breast milk. In a wealthy country such as Finland, the cohort study from Hakola et al. [76] in 2017, including 3807 mother–child dyads, found no relationship between maternal triglyceride DHA intake during the third trimester and the risk of childhood obesity between 2 and 7 years of age, although the risk appeared non-significantly lower among children in families with low maternal intake. When considered together, these findings suggest that factors such as maternal genetic profile, nutritional status, and gestational length may impact how prenatal DHA supplementation influences metabolic and genomic responses, and such effects may not be consistently seen in subsequent childhood outcomes, highlighting the need for a more personalized approach to nutrition intervention.

#### 3.4.4. Maternal DHA Supplementation and Its Impact on Infant Immunity

We identified two available studies (both conducted in Mexico) on prenatal DHA supplementation and specific immunological outcomes [50,58]. In the study by Escamilla-Nuñez et al. [58], infants were followed up to 18 months to investigate the impact of DHA 400 mg/day from week 18 of gestation until delivery on respiratory symptoms. In atopic mothers’ offspring, supplementation was associated with a 22% decrease in phlegm with nasal congestion (Incidence Rate Ratio [IRR] = 0.78; 95% CI: 0.60, 1.02) and a 47% decrease in fever with phlegm and nasal congestion (IRR = 0.53; 95% CI: 0.29, 0.99), without any impact on other respiratory symptoms. Conversely, children born to non-atopic mothers appeared to show a non-significant increase in some symptoms. DHA intake was low in this population (median: 55 mg/day; Interquartile Range [IQR]: 37–99 mg/day).

In a second study in Mexico [50], prenatal arsenic exposure was analyzed for its effects on the efficacy of DHA supplementation, and an interaction was found with the risk of atopic dermatitis (AD). DHA supplementation modified the relationship between arsenic exposure and AD (adjusted odds ratio [OR] = 1.12; 95% CI: 0.99, 1.26), with a marginal association detected in the placebo group, in which the odds were 20% higher (OR = 1.20; 95% CI: 0.70, 1.91 to IQR increase in arsenic) while a 9% reduction in the odds (OR = 0.91; 95% CI: 0.56, 1.48) was noted in the DHA group. At all follow-up points, incidence rates of AD were consistently lower in the DHA group (8.0%) than in the placebo group (8.9%) but did not achieve statistical significance. Moreover, maternal urinary arsenic concentrations (geometric mean: 28.9 µg/L; 95% CI: 24.7, 33.7) were above the international reference limits (>10 µg/L). Overall, these results suggest that prenatal DHA supplementation can decrease respiratory symptoms in infants at risk for atopy and can counteract the detrimental immunological impacts of environmental exposures.

#### 3.4.5. Maternal DHA Supplementation and Neurodevelopmental Outcomes

Evidence on maternal supplementation with DHA and the neurodevelopment of offspring has been provided from narrative reviews and RCTs. The review by de Matos Reis et al. [68], which primarily includes studies from high-income nations, concludes that adequate intake of DHA and EPA in pregnancy relates with enhanced cognitive and communicative outcomes in offspring from birth until the first 4 years of life, and that DHA and EPA deficiency is implicated for preterm delivery and uterine growth restriction as well as deficits in myelination and visual acuity. This included clinical trials and demonstrated that supplementation with 200 mg/day from week 20 of gestation improves neurodevelopment at 5 years of age, and that higher doses (600 mg/day) are associated with higher total and regional brain volume and with benefits in functions such as spatial memory and inhibitory control.

In contrast, evidence from isolated studies performed on middle-income countries, such as Mexico, shows that while no overall effects of DHA supplementation on neurocognitive development scores (subscales administered at 5 years of age) were observed, a significant interaction with FADS2 rs174602 polymorphism was found [64]. Compared to the placebo group, children of mothers receiving DHA who were also carriers of the TT genotype scored 3.5 points higher for the quantitative scale (22.6 ± 0.9 vs. 19.1 ± 0.9; *p*= 0.01, *n*= 115), and 4.3 points higher on the memory scale (27.9 ± 1.1 vs. 23.7 ± 1.1; *p* = 0.02, *n* = 115), while there were no effects for the rs174575 single nucleotide polymorphism. Moreover, Ramakris [56] enrolled 1094 pregnant Mexican women who were supplemented with 400 mg/day DHA from week 18–22 until delivery, and assessed their children’s neurodevelopment at 5 years of age in 797 participants. Although there were no significant differences in global cognition (measured with the McCarthy Scales of Children’s Abilities, MSCA, *p* = 0.64) or behavior (assessed with the Behavioral Assessment System for Children, Second Edition, BASC-2) between the two groups, those receiving the DHA supplementation performed better on sustained attention measures (Conners’ Kiddie Continuous Performance Test (K-CPT) with fewer omissions (47.6 ± 10.3 vs. 49.6 ± 11.2; *p* = 0.01), a shorter response time to the change in block (50.1 ± 12.2 vs. 52.0 ± 12.5; *p* = 0.04), and less variability in their responses (48.2 ± 10.0 vs. 49.9 ± 9.4; *p* = 0.01). There were no differences in impulsivity or general risk of ADHD; furthermore, the authors found a significant (*p*-interaction < 0.05) interaction between DHA and the quality of the home environment, suggesting that DHA may interact with environmental influences on child development [56]. These findings support the importance of accounting for differences in maternal genetics and contextual factors in designing DHA intervention strategies that would benefit child neurodevelopment.

### 3.5. DHA in Breast Milk and Infant Health Outcomes

#### 3.5.1. Maternal DHA Intake and Composition of Human Milk

Multiple studies have demonstrated that breast milk DHA content is directly impacted by maternal diet, especially fish and seafood consumption. A systematic review by Petersohn et al. [84] included 11 studies in which the most consistent predictor of higher milk DHA concentrations was maternal intake of fish and other foods providing preformed DHA; occasional correlations with non-DHA foods likely reflect overall diet quality rather than direct DHA provision.

Geographical variations have also been widely reported in cross-sectional observational studies. In Asia, Nguyen et al. [46] found the mean breast milk DHA level was 0.53% in China, 0.50% in Korea, 0.48% in Vietnam, and 0.23% in Pakistan. Similarly, Gao et al. [44], reported higher milk DHA levels (0.40%; IQR: 0.33, 0.55) in Cambodia compared with Australian levels (0.23%; IQR: 0.17–0.34; *p* < 0.0001), even though Cambodian milk had lower fat concentrations; this was attributed to the fact that 95% of Cambodian women eat fish every day, in contrast to < 20% of Australian women. In Latin America, a longitudinal study in Brazil [73] revealed that only DHA intake during the third trimester of pregnancy was significantly associated with the DHA concentration in mature milk (β = 0.464; 95% CI: 0.212, 0.714), accounting for 33% of its variability. In Chile, Barrera et al. [79] showed a decrease of 50% in the consumption of DHA values during the first and sixth months of lactation when compared to pregnancy, also translating into a decrease in DHA levels in breast milk (from 0.39% to 0.14%) and erythrocytes (from 4.16% to 3.01%). Regarding the main food groups that contribute to DHA intake, Ding et al. [42] reported in a cross-sectional study in 121 Chinese women that among four main dietary patterns in this population, the egg and aquatic product-based pattern offered the highest dietary DHA intake (73.9 ± 184.2 mg/d) and milk DHA concentrations (0.23 ± 0.17%; *p* = 0.002).

On the other hand, DHA concentrations in breast milk have also been studied in vulnerable populations such as pregnant adolescents. De Souza Santos da Costa et al. [40] reported a DHA intake in pregnant adolescents accounting for only 0.04% of total energy. The milk DHA content of this group decreased from 0.92% in colostrum to 0.17% in mature milk (*p* < 0.05); and a high *n*-6:*n*-3 ratio was associated with low fish intake and high intake of *n*-6 fatty acid-rich vegetable oils. Another population group that could be at greater nutritional risk is pregnant women with obesity, suggesting that maternal obesity has adverse effects on DHA levels. Chamorro et al. [48] reported lower concentrations of DHA in erythrocytes and breast milk from women with obesity in a longitudinal Chilean study, despite their dietary intakes being similar to those observed for eutrophic women. The authors also found that milk DHA at one month and at six months (r = −0.83 vs. r = −0.91, respectively; *p* = 0.0001) had an inverse relationship with postpartum BMI, thus indicating metabolic changes that may modify the transfer of DHA to the infant. Finally, Vizzari et al. [43] in Italy assessed the milk content of 103 mothers of full-term newborns with 88 mothers of premature infants and identified significantly lower concentrations of DHA in the milk of mothers of premature infants (0.51% vs. 0.71%; *p* = 0.001); 37% of these women did not achieve the European Food Safety Authority DHA recommendations compared to full-term mothers (20%). These findings suggest that the levels of DHA in breast milk are affected by maternal diet, physiology, and other contextual determinants, such as income and cultural practices. Awareness of this variability is crucial for tailoring effective, context-specific nutritional interventions in lactation.

#### 3.5.2. DHA in Breast Milk and Child Health Outcomes

Associations of human milk DHA content with child developmental outcomes have been described in several studies, particularly in the neurological, motor, immunological, and growth domains. For example, RBC-DHA levels were significantly correlated with movement pattern assessments in an observational study conducted in Tanzania and the Netherlands, explaining 16% of the variability in observed movement patterns [47], indicating a link between higher DHA status and better motor development in early childhood. Additionally, Mallick et al. [3] observed in a review that DHA is critical for fetal brain development and is positively associated with cognitive assessments such as IQ and problem solving among typically developing children. Martins et al. [70] also found that higher maternal DHA during pregnancy and lactation was correlated with lower rates of ADHD, indicating a protective role of DHA against hyperactivity and inattention in childhood.

With respect to the immune system, in a narrative review, Richard et al. [16] examined the role of DHA in the early development of the immune system, finding that lower concentration of DHA in breast milk at three months postpartum was associated with a higher incidence of atopic diseases at 18 months compared with non-atopic infants (0.19% ± 0.09% vs. 0.25% ± 0.16%). Lower DHA concentrations were detected in the milk of mothers whose children developed atopic dermatitis between 3 and 6 months of age, and a significant inverse association was found between DHA content and asthma risk at the age of 4 years among the children of mothers with allergic disease history [16]. Moreover, higher plasma DHA levels were associated with lower IL-13 and IL-5 production on stimulation with β-lactoglobulin, while low plasma DHA levels predicted eczema before the age of 1 year [16]. The review carried out by Falize et al. [82], reinforces these findings, indicating that three cohort studies have shown lower DHA concentrations in the milk of mothers with atopic dermatitis or allergic symptoms, and one study reported an inverse association between DHA in breast milk and asthma diagnosis at age 4 in infants of mothers with atopy (OR = 0.39; 95% CI: 0.16, 0.99). They also reported a positive correlation between increased DHA levels and decreased allergic sensitization, as well as an inverse correlation between plasma concentrations of DHA and IL-13 and IL-5 production, supporting the potential immune regulatory role of DHA [82]. These results support the neurodevelopmental and immunomodulatory effects of DHA in infancy. Higher levels of DHA in breast milk are consistently linked to better motor, cognitive, and behavioral outcomes and a reduced risk of allergic disease in early childhood.

### 3.6. Strengths and Limitations

To interpret the findings of this review, several methodological limitations must be acknowledged. The broad heterogeneity of study designs, populations, outcome measures, and DHA exposure assessments complicates comparability and precludes quantitative synthesis or meta-analysis. Most of the included evidence is based on small-scale or localized populations, and the lack of nationally representative dietary surveys in low- and middle-income countries restricts the ability to estimate the prevalence of DHA deficiency with accuracy. Furthermore, differences in dietary assessment instruments and biomarker quantification methods reduce precision in linking exposure with outcomes. Although Table 1 specifies when studies reported adjustments for potential confounders, not all studies applied these controls, which may influence the reported associations. Finally, as this was conceived as a narrative review, a formal risk of bias assessment was not performed; thus, future systematic reviews and meta-analyses are needed to provide pooled estimates.

This review includes 76 studies from 2014 to 2025 and applies a structured methodology based on the PICOS framework. Its strengths lie in the comprehensive synthesis of evidence from observational, interventional, and systematic reviews and meta-analyses, with an explicit focus on low- and middle-income countries. The review also identifies critical disparities in intake patterns across geographic and income strata, emphasizing the need to address deep inequalities in access to nutrition among vulnerable populations. Given the heterogeneity of study designs, populations, exposures, and outcomes, evidence was organized and interpreted by life stage and by national income level to improve comparability.

## 4. Conclusions and General Considerations for Public Health

This narrative review contributes to the evidence that reinforces the role of DHA as an essential nutrient throughout early life, due to its essential roles in neurodevelopment, immune regulation, and the maintenance of physiological functions, as well as in health outcomes later in life. However, DHA intake remains persistently insufficient during pregnancy, lactation, infancy, and childhood in low- and middle-income countries (LMICs), with implications for equity in child health and early development.

DHA deficiency in LMICs cannot be attributed solely to limited access to marine sources. Multiple structural, cultural, and systemic factors, repeatedly described in the included literature, may contribute to low DHA intake. These include the predominance of plant-based dietary patterns that are low in DHA; limited integration of DHA into national dietary guidelines; inconsistent or nonexistent supplementation policies targeting pregnant and lactating women; cost-related barriers; and the limited availability of fortified foods or products containing DHA. Importantly, only fish and seafood, eggs, and selected animal products provide meaningful amounts of preformed DHA, while plant-based foods contribute mainly alpha-linolenic acid, whose conversion to DHA is highly inefficient. Furthermore, sociocultural preferences, regional disparities in food availability and accessibility, and the exclusion of DHA-rich products from social support programs can exacerbate these challenges.

The findings suggest that dietary DHA intake or supplementation is associated with improved outcomes in neurodevelopment, the immune system, pregnancy, and blood pressure; however, the evidence supporting its effects on blood pressure is limited and should be interpreted cautiously. Nevertheless, the inconsistencies found in the results of some studies, often due to gene–environment interactions, baseline nutritional status, or the timing of interventions, together with differences in dietary assessment tools and in whether studies adjusted for confounders, highlight the need for future research with more specific and well-defined approaches and contexts. Standardized dietary assessment methods, consistent adjustment for key confounding factors, and regionally or genetically informed designs will be necessary to strengthen comparability. Moreover, future systematic reviews and meta-analyses are needed to provide pooled estimates that complement the narrative evidence.

Overall, this review highlights the importance of recognizing DHA deficiency as a broad public health problem during early childhood and not merely as a shortfall in individual intake. This review underscores the need to generate nationally representative data to understand population DHA intake across countries, develop culturally acceptable, affordable, and context-appropriate dietary policies, and invest in population-based strategies for DHA administration through supplementation or food fortification. Nutrition interventions should consider regional consumption patterns, socioeconomic barriers, and systemic inequalities that limit access to DHA in early childhood. Addressing these factors comprehensively is essential to promote child health equity in low- and middle-income countries.

## Data Availability

No new data were created or analyzed in this study.

## Supplementary Materials

The following supporting information can be downloaded at: https://www.mdpi.com/article/10.3390/nu17243931/s1, Supplemental Table S1. Classification of DHA-Related Outcomes in Neurodevelopment, General Development, and Immune Function in Children. Supplemental Table S2. World Bank classification of countries by Gross National Income (GNI) per capita, fiscal year 2023 (Atlas method). Supplemental Figure S1. PRISMA 2020 flow diagram of the literature search and study selection process for the narrative review on dietary DHA intake and supplementation from pregnancy through childhood.

## Abbreviations

The following abbreviations are used in this manuscript:

ADHD	Attention-Deficit/Hyperactivity Disorder
ADORE	Assessment of DHA on Reducing Early Preterm Birth (clinical trial)
ALA	Alpha-linolenic Acid
ARA	Arachidonic Acid
BMI	Body Mass Index
CC	Homozygous Genotype (context-dependent)
CI	Confidence Interval
DDE	1,1-dichloro-2,2-bis (*p*-chlorophenyl)ethylene
DHA	Docosahexaenoic Acid
DMR	Differentially Methylated Region
DP	Dietary Patterns
DXA	Dual-Energy X-ray Absorptiometry
ENSANUT	Mexican National Health and Nutrition Survey
EPA	Eicosapentaenoic Acid
FADS	Fatty Acid Desaturase
FAO	Food and Agriculture Organization
FBS	Food Balance Sheets
FDR	False Discovery Rate
GEE	Generalized Estimating Equations
GNI	Gross National Income
HDL	High-Density Lipoprotein
HICs	High-Income Countries
HOME	Home Observation for Measurement of the Environment
IQ	Intelligence Quotient
IQR	Interquartile Range
IRR	Incidence Rate Ratio
KABC	Kaufman Assessment Battery for Children
LA	Linoleic Acid
LCPUFA	Long-Chain Polyunsaturated Fatty Acids
LDL	Low-Density Lipoprotein
LMICs	Low- and Middle-Income Countries
MeSH	Medical Subject Headings
MSCA	McCarthy Scales of Children’s Abilities
NDSR	Nutrition Data System for Research
NAFLD	Non-Alcoholic Fatty Liver Disease
OR	Odds Ratio
PCA	Principal Component Analysis
PICOS	Population, Intervention, Comparison, Outcome, Study Design
PPVT	Peabody Picture Vocabulary Test
PRISMA	Preferred Reporting Items for Systematic Reviews and Meta-Analyses
PTB	Preterm Birth
PUFAs	Polyunsaturated Fatty Acids
RBC	Red Blood Cells
RCT	Randomized Controlled Trial
RNI	Reference Nutrient Intake
SPTB	Spontaneous Preterm Birth
TC	Total Cholesterol
TCA	Tricarboxylic Acid
TNF	Tumor Necrosis Factor
TT	Homozygous Genotype (for T allele)
USDA	United States Department of Agriculture
WHO	World Health Organization
