# Supplementary material for: Dietary Patterns of Docosahexaenoic Acid Intake and Supplementation from Pregnancy Through Childhood with a Focus on Low- and Middle-Income Countries: A Narrative Review of Implications for Child Health
Source: Nutrients. 2025 Dec 16;17(24):3931. doi: 10.3390/nu17243931 (PMC12735697; doi:10.3390/nu17243931)
Supplement: Supplementary file 1 [file nutrients-17-03931-s001.zip › nutrients-3939447-supplementary.pdf]

nutrients

Review

Dietary Patterns of Docosahexaenoic Acid Intake and Supplementation from Pregnancy through Childhood with focus on Low- and Middle-Income Countries: A Narrative Review of Implications for Child Health

Brenda Valle-Valdez [1], Xochitl Terrazas-López [2]; Alejandra González-Rocha [3], Humberto Astiazarán-García [4] and Brianda Armenta-Guirado [5,*]

SUPPLEMENTAL MATERIAL

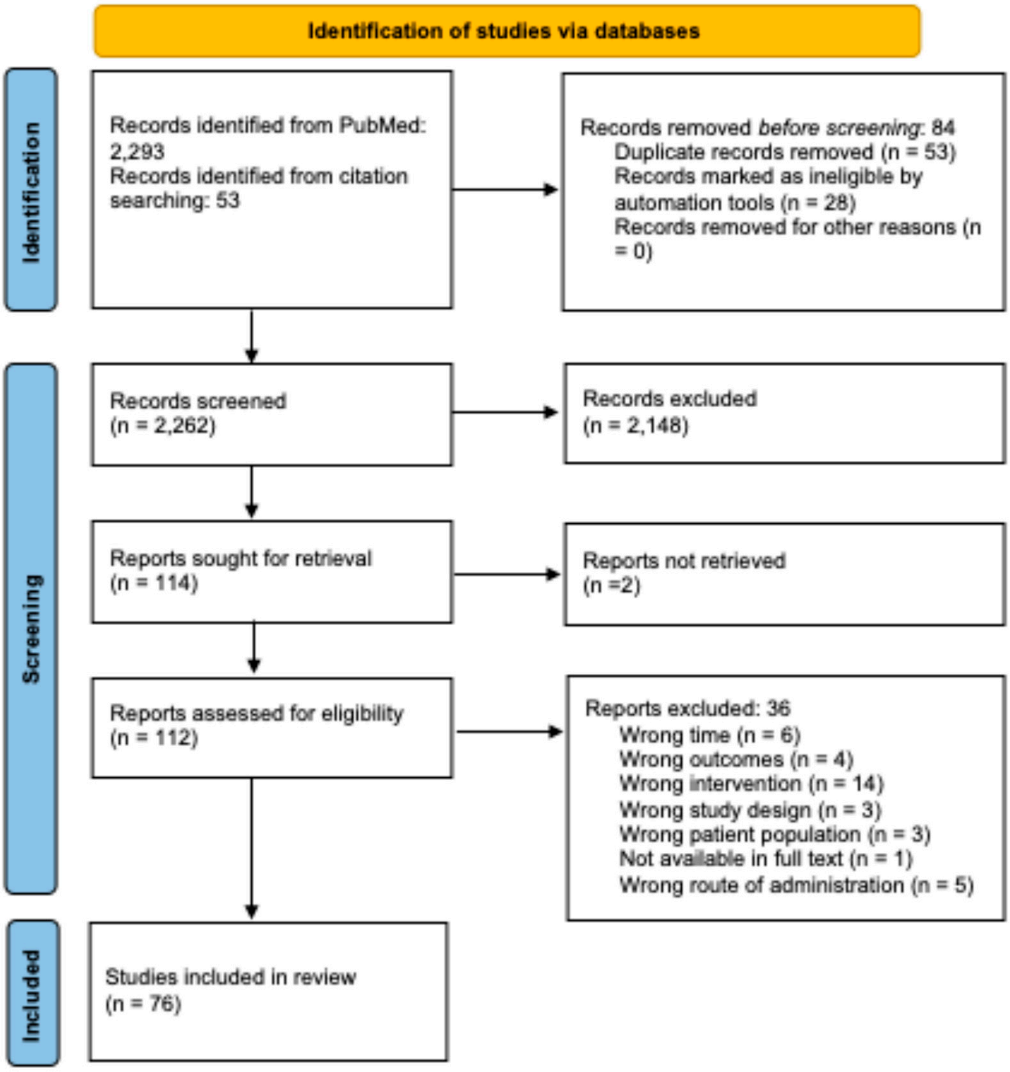

Supplemental Figure S1. PRISMA 2020 flow diagram of the literature search and study selection process for the narrative review on dietary DHA intake and supplementation from pregnancy through childhood.

Supplemental Table S1. Classification of DHA-Related Outcomes in Neurodevelopment, General Development, and Immune Function in Children.

Outcome	Neurodevelopment	General Development	Immune System
Cognition and learning	✓	X	X
Language and communication	✓	X	X
Attention and memory	✓	X	X
Fine motor skills	✓e.g., eye-hand coordination)	X	X
Gross motor skills	X	✓ (e.g., crawling, walking, balance)	X
Physical growth	X	✓ (e.g., weight, height, head circumference)	X
Cardiovascular function	X	✓ (e.g., blood pressure)	X
Pulmonary maturation	X	✓	X
Body composition	X	✓ (e.g., lean mass, BMI)	X
Inflammation reduction	X	X	✓ (e.g., lower IL-6, TNF-α levels)
Lower risk of atopic diseases	X	X	✓ (e.g., lower risk of atopic dermatitis, asthma)
Improved immune response	X	X	✓ (e.g., increased antibody production, reduced infection susceptibility)

✓ indicates inclusion in the domain

X indicates exclusion.

This classification synthesizes the outcomes identified in the present study for organizational purposes, based on current scientific evidence on DHA in children.

Supplemental Table S2. World Bank classification of countries by Gross National Income (GNI) per capita, fiscal year 2023 (Atlas method).

GNI Classification	Gross National Income (GNI) per capita (USD, 2022 Atlas method)
Low income	≤ 1,135
Lower middle income	1,136 – 4,465
Upper middle income	4,466 – 13,845
High income	≥ 13,846

Country income groups are based on the World Bank classification for fiscal year 2023.

Thresholds are determined using the Atlas method, which smooths exchange rate fluctuations by applying a three-year moving average, price-adjusted conversion factor.

Classification is based on 2022 GNI per capita estimates.
